# Dataset on the performance of Atlantic salmon (*Salmo salar*) reared at different dissolved oxygen levels under experimental conditions

**DOI:** 10.1016/j.dib.2024.110983

**Published:** 2024-09-28

**Authors:** Nina Liland, Ivar Rønnestad, Marina Azevedo, Floriana Lai, Frida Oulie, Luís Conceição, Filipe Soares

**Affiliations:** a Institute of Marine Research, P.O. Box 1870, Nordnes, Bergen 5817, Norway; b Department of Biological Sciences, University of Bergen, Bergen 5006, Norway; c Sparos Lda., Área Empresarial de Marim, Lote C, 8700-221 Olhão, Portugal

**Keywords:** Aquaculture, Feed intake, Fish farming, Growth, Salmonids, Water parameters

## Abstract

Atlantic salmon (*Salmo salar*) cultivated in cages and net-pens are regularly exposed to natural variations in dissolved oxygen levels, occasionally experiencing events of low oxygen availability. Quantifying the impact of low dissolved oxygen levels on fish performance can help fish farmers better manage the risks associated with such events.

This article describes the zootechnical performance of Atlantic salmon reared under experimental conditions at three different dissolved oxygen levels (i.e., low: 50 % saturation; medium: 60 % saturation; high: 95 % saturation). The data was collected in the context of two in vivo trials: (i) Trial A, where fish with an initial average body weight of 312.44 ± 11.53 g were reared in indoor tanks at the different DO levels for 30 days; (ii) Trial B, where fish with an initial average body weight of 735.33 ± 40.42 g were reared in indoor tanks at the different DO levels for 26 days.

The dataset [1] is composed of spreadsheets (.xlsx format) and charts (.png format), and includes daily and hourly resolution data (e.g., dissolved oxygen, water temperature, salinity, number of fish and feed intake), sampling and laboratory data (e.g., fish weight, fork length, sex, organs weight, whole-body composition, and tail and opercular beat frequency), and zootechnical indicators calculated at the tank level and averaged per treatment (e.g., survival rate, weight gain, cumulative feed intake, feed conversion ratio and somatic indexes). The differences between treatment means were analyzed using ANOVA, followed by post-hoc testing.

The data presented here has the potential to be used in subsequent analyses, for example when analyzed together with other experimental data or through its use to parameterize mathematical models, aiming at better understand and describe the effects of dissolved oxygen on the performance of Atlantic salmon.

Specifications Table

**Table:** 

Subject	Agricultural sciences (Aquaculture)
Specific subject area	Influence of environmental parameters on the zootechnical performance of fish
Type of data	Spreadsheets (.xlsx format): including Raw, Processed and Analyzed data Charts (.png format)
Data collection	Hourly and/or daily resolution data: Water temperature and dissolved oxygen (measured by sensors); salinity (measured by sensors); number of fish (visually inspected and counted manually); feed intake (estimated by difference based on the feed provided and recovered in each tank, measured using a weighing scale). Sampling and laboratory data: fish and organs weight (measured using a weighing scale); fork length (manually measured using a ruler); sex (visually identified); whole-body composition (measured following AOAC methods); tail and opercular beat frequency (video footage and analysis). Zootechnical indicators: Calculated based on daily resolution and sampling data.
Data source location	Facilities: Matre Research Station, Institute of Marine Research (IMR) Address: 382 Matre 5, 5984 Matredal, Norway Coordinates: 60.87413° N and 5.58564° E)
Data accessibility	Repository name: Mendeley Data Data identification number: doi:10.17632/b9v6tsg3fh.1 Direct URL to data: https://data.mendeley.com/datasets/b9v6tsg3fh/1
Related research article	None

## 1 Value of the Data

• This dataset contains quantitative measurements of Atlantic salmon reared over a short period (1 month), under experimental conditions, at different dissolved oxygen levels (50 %, 60 % and 95 % saturation).
• It can contribute to a better understanding of the effects of dissolved oxygen on Atlantic salmon performance.
• It provides quantitative information that can help fish farmers better manage the risks associated with low oxygen events.
• It can be used as a reference to support the experimental design of future studies, as well as a reference in applications for obtaining approval from animal welfare authorities to carry out experimental studies with fish reared at low dissolved oxygen levels.
• It can be used, together with other data, in further analyses aiming at better describe the effects of dissolved oxygen on Atlantic salmon performance (for example, to support the development of mathematical models) [1].

## 2 Background

Atlantic salmon (*Salmo salar*) farmed in sea cages can experience periods of low oxygen availability [[2], [3], [4]]. Quantifying the response of salmon to decreasing dissolved oxygen (DO) levels is crucial, as it can help salmon farmers better manage the risks associated with these events. Previous studies have measured salmon responses to short-term cyclic DO variations. For instance, one study exposed salmon to hypoxia conditions (50 % DO saturation) for one hour every six hours [5], while another subjected them to random variations in DO saturation (seven levels ranging from 32 % to 122 %) every second day [6].

However, salmon may exhibit conformism behavior, where their metabolic rate decreases with decreasing oxygen levels [7]. This suggests that the response magnitude to short-term DO variations (e.g., four times a day or every other day) may differ from the response to prolonged low oxygen levels (e.g., one month). Here, we present a dataset that includes measurements of the zootechnical performance of salmon reared under three different constant DO levels for one month.

## 3 Data Description

**Fig. 1:**
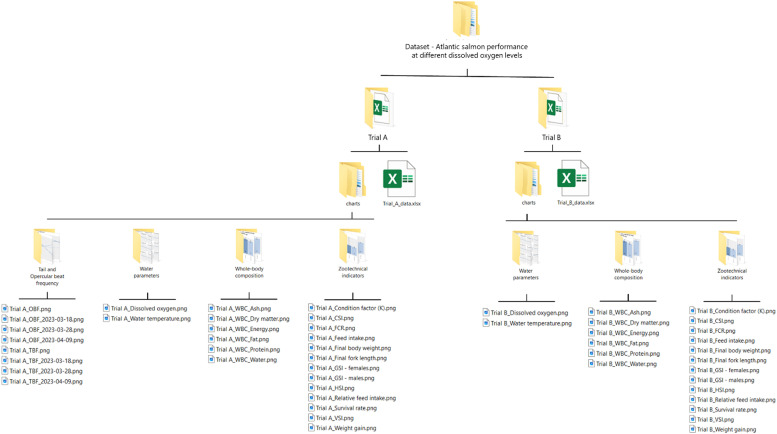
Structure of the ‘Dataset – Atlantic salmon performance at different dissolved oxygen levels’ folder.

Fig. 1 describes the structure of the ‘Dataset – Atlantic salmon performance at different dissolved oxygen levels’ folder.

The main folder comprises two sub-folders, each corresponding to a trial, e.g., 'Trial A' and 'Trial B'. These sub-folders are organized similarly and encompass trial-specific data. Nonetheless, Trial A provides supplementary information on tail and opercular beat frequency, which is absent for Trial B. In essence, each sub-folder encompasses:

■ A spreadsheet either with 14 sheets (‘Trial_A_data.xlsx’) or 11 sheets (‘Trial_B_data.xlsx’), containing hourly and daily resolution data, sampling and laboratory data, and zootechnical indicators at both the tank and treatment level.
■ The ‘charts’ folder contains .png format charts generated using the R software. These include time series of water parameters with daily resolution at the tank level, bar charts depicting whole-body composition, zootechnical indicators, and tail and opercular beat frequency at the treatment level, and line charts illustrating the variation of tail and opercular beat frequency over time at the treatment level. The bar charts display averages, standard deviations (SD), and superscript letters indicating statistically significant differences between treatments.

Table 1 provides detailed information on the data included in each sheet of the spreadsheet files (‘Trial_A_data.xlsx’ and ‘Trial_B_data.xlsx’). Additionally, it explains the correspondence between the data in the spreadsheet files (.xlsx format) and the charts (.png format).

**Table 1:** Description of the data available in the .xlsx file and correspondence with .png files.

Sheet name	Description	Correspondence with .png files available in the ‘charts’ folder*
INFO	Includes metadata about the trial: • trial name; • trial description; • start and end date; • species; • location; • list tanks and treatments.	–
DietComposition	Includes data on diet composition analysis for: • crude protein [% as fed]; • crude lipids [% as fed]; • ash content [% as fed]; • gross energy [MJ/kg as fed]; • phosphorus [% as fed]; • amino acid profile [% as fed].	–
WaterParameters Hourly	Includes time series data (with hourly resolution) at the tank level for the following recorded water parameters: • water temperature [°C]; • salinity [ppt]; • dissolved oxygen [% saturation].	–
WaterParameters Daily	Includes time series data (with daily resolution) at the tank level for the following recorded water parameters: • water temperature [°C]; • salinity [ppt]; • dissolved oxygen [% saturation and mg/L].	Sub-folder: *Water parameters* File names: *Trial ID_Dissolved oxygen.png* *Trial ID_Water temperature.png*
NumberOfFish	Includes time series data (with daily resolution) at the tank level for: • total number of fish; • number of dead fish.	–
FeedIntake	Includes time series data [with daily resolution] at the tank level for: • individual daily feed intake [g/fish]; • individual cumulative feed intake [g/fish].	–
Samplings	Includes samplings and laboratory data at the tank and replicate level. The table includes the following columns: • Date; • Tank; • Replicate; • Sample type; • Fish weight [g]; • Fork length [cm]; • Sex; • Liver weight [g]; • HSI [%]; • Heart weight [g]; • CSI [%]; • Gonad weight [g]; • GSI [%]; • Viscera weight [g] • VSI [%].	–
TailOpercularBeat_ records⁎⁎	Includes data on tail and opercular beat frequency at the fish level. The table includes the following columns: • Date; • Treatment; • Tank; • Record; • Fish; • Tail beat frequency (TBF) [beats/second]; • Opercular beat frequency (OBF) [beats/second].	–
TailOpercularBeat_ tank⁎⁎	Includes data on tail and opercular beat frequency at the tank level. The table includes the following columns: • Date; • Treatment; • Tank; • Metric; • Tail beat frequency (TBF) [beats/second]; • Opercular beat frequency (OBF) [beats/second].	–
TailOpercularBeat_ treatment⁎⁎	Includes data on tail and opercular beat frequency at the treatment level. The table includes the following columns: • Date; • Treatment; • Metric; • Tail beat frequency (TBF) [beats/second]; • Opercular beat frequency (OBF) [beats/second].	Sub-folder⁎⁎: *Tail and Opercular beat frequency* File names⁎⁎: *Trial A_OBF.png* *Trial A_OBF_2023-03-18.png* *Trial A_OBF_2023-03-28.png* *Trial A_OBF_2023-04-09.png* *Trial A_TBF.png* *Trial A_TBF_2023-03-18.png* *Trial A_TBF_2023-03-28.png* *Trial A_TBF_2023-04-09.png*
BodyComposition_ tank	Includes whole-body composition data at the tank level. The table includes the following columns: • Date; • Treatment; • Tank/Replicate; • Dry matter [g/100 g]; • Water [g/100 g]; • Ash [g/100 g]; • Energy [kJ/g]; • Fat [g/100 g]; • Protein [g/100 g]; • Ca [mg kg^-1^]; • K [mg kg^-1^]; • Mg [mg kg^-1^]; • Na [mg kg^-1^]; • Phosphorus [mg kg^-1^].	–
BodyComposition_ treatment	Includes whole-body composition data at the treatment level. The table includes the following columns: • Date; • Treatment; • Metric; • Dry matter [g/100 g]; • Water [g/100 g]; • Ash [g/100 g]; • Energy [kJ/g]; • Fat [g/100 g]; • Protein [g/100 g]; • Ca [mg kg^-1^]; • K [mg kg^-1^]; • Mg [mg kg^-1^]; • Na [mg kg^-1^]; • Phosphorus [mg kg^-1^].	Sub-folder: *Whole-body composition* File names: *Trial ID_WBC_Ash.png* *Trial ID_WBC_Dry matter.png* *Trial ID_WBC_Energy.png* *Trial ID_WBC_Fat.png* *Trial ID_WBC_Protein.png* *Trial ID_WBC_Water.png*
ZootechnicalTable_ tank	Includes zootechnical indicators at the tank level. The table includes the following columns: • Tank; • Treatment; • Initial date; • Final date; • Average dissolved oxygen [mg/L]; • Initial number of fish; • Final number of fish; • Survival rate [%]; • Initial body weight [g]; • Final body weight [g]; • Weight gain [g/fish]; • Final fork length [cm]; • Condition factor [K]; • Feed intake [g/fish]; • Relative feed intake [% weight/day]; • FCR [g feed/g weight gain]; • HSI [%]; • CSI [%]; • GSI – females [%]; • GSI – males [%]; • VSI [%].	–
ZootechnicalTable_ treatment	Includes zootechnical indicators at the treatment level. The table includes the following columns: • Treatment; • Metric; • Initial date; • Final date; • Average dissolved oxygen [mg/L]; • Initial number of fish; • Final number of fish; • Survival rate [%]; • Initial body weight [g]; • Final body weight [g]; • Weight gain [g/fish]; • Final fork length [cm]; • Condition factor [K]; • Feed intake [g/fish]; • Relative feed intake [% weight/day]; • FCR [g feed/g weight gain]; • HSI [%]; • CSI [%]; • GSI – females [%]; • GSI – males [%]; • VSI [%].	Sub-folder: *Zootechnical indicators* File names: *Trial ID_Condition factor (K).png* *Trial ID_CSI.png* *Trial ID_FCR.png* *Trial ID_Feed intake.png* *Trial ID_Final body weight.png* *Trial ID_Final fork length.png* *Trial ID_Final fork length.png* *Trial ID_GSI – females.png* *Trial ID_GSI – males.png* *Trial ID_HSI.png* *Trial ID_Relative feed intake.png* *Trial ID_Survival rate.png* *Trial ID_VSI.png* *Trial ID_Weight gain.png*

⁎ The nomenclature of the .png file names [charts) includes a prefix that references the trial. In this table, the trial reference is defined as ‘ID,’ which indicates either ‘A’ or ‘B.’.

⁎⁎ Sheets, folders, and files only available for Trial A.

## 4 Experimental Design, Materials and Methods

### 4.1 Experimental design

**Fig. 2:**
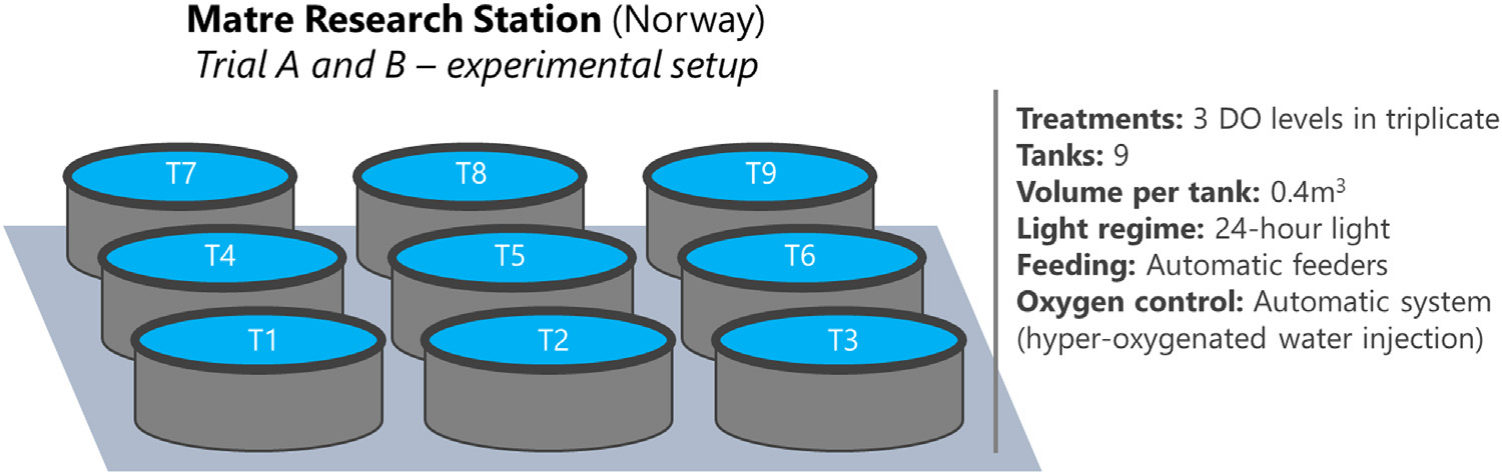
Experimental setup in Trial A and B.

Two *in vivo* trials, A and B, were carried out with Atlantic salmon (*Salmo salar*) at the Matre Research Station (Matre, Norway) under experimental scale conditions, following the same experimental design, consisting of three treatments (different dissolved oxygen levels) tested in triplicate tanks (see Table 2 and Fig. 2). The main difference between trials is related with the size of the fish. Trial A and B were carried out with post-smolt salmon with initial weights at around 300 g and 700 g, respectively. Due to restrictions in biomass per tank, the number of fish per tank were higher in trial A than in trial B. The two trials lasted for 30 and 26 days, respectively, the latter trial being a few days shorter due to bank holidays and access to trained personnel for sampling of tissue for other tasks in the project. Due to less access to staff during some weekends and holidays, there are some gaps in the feed intake data. This can be managed when using the data by, e.g. estimating feed intake for those missing days based on the feed intake registered the days immediately before and after the missing data.

**Table 2:** Experimental treatments tested in Trial A and B, consisting of three different dissolved oxygen (DO) target levels. Number of replicate tanks per oxygen level indicated by n.

Treatment	n	DO target level
DO50 % (low oxygen level)	3	50 % saturation
DO60 % (medium oxygen level)	3	60 % saturation
DO95 % (high oxygen level)	3	95 % saturation

### 4.2 Fish rearing and samplings

#### 4.2.1 Trial A

Trial A was carried out from 16 to 03–2023 to 14–04–2023. Nine (9) indoor tanks (experimental units: T1, T2, T3, T6, T7, T8, T9, T10, T11; volume: 0.4 m^3^; light regime: 24 L) were stocked with 45 individuals each (average initial body weight of 312.44 ± 11.53 g). The dissolved oxygen levels were adjusted by controlling the water flow rate and by adding hyper-oxygenated water based on the targets defined in Table 2 (automatic oxygen control system).

The water parameters varied as follows: temperature 12.00 ± 0.23 °C; dissolved oxygen 4.95 ± 0.39 mg/L (53.18 ± 1.19 % saturation; DO50 %), 5.68 ± 0.14 mg/L (60.89 ± 0.52 % saturation; DO60 %), 8.89 ± 0.01 mg/L (95.00 ± 0.00 % saturation; DO95 %); salinity 22.07 ± 0.10 ppt.

Fish were fed a single diet (Protec, Skretting; 47 % crude protein, 22 % crude lipids, 22 kJ/g gross energy; 4.5 mm pellet size), two meals a day, with automatic feeders (from 09:00–11:00 and 13:00–15:00). Feeding was done in excess and adjusted regularly to appetite. Feed spill was collected with a sieve from the outlet water within 15 min after each feeding. The conversion factor from wet-to-dry feed (recovery coefficient, Rc) was assessed by adding a weighed amount of feed to a tank without fish in the same technical line-up and drying the collected feed spill to assess water retention. This was repeated twelve times to get representative measurements. The following formula was used to estimate the recovery coefficient: Rc =*W*/A, where W is the wet weight (g) of the waste feed collected and A is the dry weight (g) of pellets supplied to the tank. The total feed provided and recovered in each tank was recorded twice on a daily basis.

At day 1 (16-03-2023), total biomass and total number of fish in each tank were measured to estimate the initial average body at the tank level, 9 fish were individually weighed and fork length measured. At day 30 (14-04-2023) fork length and weight were measured (with and without viscera) of all fish in the tanks. Additionally, samples were taken for, analyses of whole-body (3 fish pooled into one (1) sample per tank) and viscera/carcass composition (6 fish per tissue, divided into two (2) pooled samples per tank). For 10 fish per tank, gender was identified and heart, liver and gonads weighed. Moreover, on day 3 (18–03–2023), day 13 (28-03-2023), and day 25 (09-04-2023), underwater footage was captured to gather video recordings for the analysis of tail and opercular beat frequency.

#### 4.2.2 Trial B

Trial B was carried out from 15 to 05–2023 to 09–06–2023. Nine (9) indoor tanks (experimental units: T1, T2, T3, T6, T7, T8, T9, T10, T11; volume: 0.4 m^3^; light regime: 24 L) were stocked with 20 individuals each (average initial body weight of 735.33 ± 40.42 g). The dissolved oxygen levels were adjusted by controlling the water flow rate and by adding hyper-oxygenated water, based on the targets defined in Table 2 (automatic oxygen control system).

The water parameters varied as follows: temperature 12.07 ± 0.12 °C; dissolved oxygen 5.14 ± 0.06 mg/L (55.10 ± 0.73 % saturation; DO50 %), 5.90 ± 0.04 mg/L (63.26 ± 0.47 % saturation; DO60 %), 8.86 ± 0.02 mg/L (94.84 ± 0.20 saturation; DO95 %); salinity 22.0 ± 0.25 ppt.

Fish were fed a single diet (Protec, Skretting; 46 % crude protein, 25 % crude lipids, 23 kJ/g gross energy; 4.5 mm pellet size), two meals a day, with automatic feeders (from 09:00–11:00 and 13:00–15:00). Feeding was done in excess and adjusted regularly to appetite. Feed spill was collected with a sieve from the outlet water within 15 min after each feeding. The conversion factor from wet-to-dry feed (recovery coefficient, Rc) was assessed by adding a weighed amount of feed to a tank without fish in the same technical line-up and drying the collected feed spill to assess water retention. This was repeated twelve times to get representative measurements. The following formula was used to estimate the recovery coefficient: Rc =*W*/A, where W is the wet weight (g) of the waste feed collected and A is the dry weight (g) of pellets supplied to the tank. The total feed provided and recovered in each tank was recorded twice on a daily basis.

At day 1 (15-05-2023), total biomass and total number of fish in each tank were measured to estimate the initial average body at the tank level, and 9 fish were individually weighed and fork length measured. At day 30 (09-06-2023) fork length and weight were measured (with and without viscera) of all fish in the tanks. Additionally, samples were taken for, analyses of whole-body (3 fish pooled into one (1) sample per tank) and viscera/carcass composition (6 fish per tissue, divided into two (2) pooled samples per tank). For 10 fish per tank, gender was identified and heart, liver and gonads weighed.

### 4.3 Analytical methods

At the beginning of each the trial, fish weight was estimated in bulk using a weighing scale. At the end of each the trial, fish and organ weight were estimated at the individual level using a weighing scale, and fork length was estimated at the individual level using a ruler. Homogenized whole-body and carcass/viscera samples were analyzed for their content of dry matter, fat, protein (nitrogen) and ash. For dry matter, samples were freeze-dried (freezing for 24 h, at −20 °C in vacuum, 0.2–0.01 mBar, followed by vacuum at 25 °C until constant weight was reached). Total fat was determined gravimetrically after ethyl-acetate extraction. Total nitrogen was analyzed using a CHNS elemental analyzer (Vario Macro Cube; Elementar Analysensysteme GmbH, Langenselbold, Germany) and quantified by using the conversion factor of 6.25, according to official AOAC guidelines [8]. Ash content was determined by combustion at 750 °C using the automated macro thermogravimetric analyser, Leco TGA 801 (Leco, St. Joseph, MI, USA).

### 4.4 Zootechnical indicators

Zootechnical indicators, such as the survival rate (SR), weight gain (WG), feed intake (FI), relative feed intake (RFI), feed conversion ratio (FCR), condition factor (K), hepatosomatic index (HSI), cardiosomatic index (CSI), gonadosomatic index (GSI) and viscerosomatic index (VSI), were estimated at the tank level and then averaged per treatment. These indicators were calculated as follows:

Survival rate (SR)

(1) $$SR\;=\frac{initial_{fish}-dead_{fish}}{initial_{fish}}\times \;100$$

where $initial_{fish}$ is the initial number of fish and $dead_{fish}$ is the number of fish that died in the period.

Weight gain (WG)

(2) WG=FBW−IBW

where WG is the weight gain (g), FBW is the final body weight (g) and IBW is the initial body weight (g).

Feed intake (FI)

(3) $$FI\;=\sum_{i=1}^{m}\frac{FeedProvided_{i}-FeedRecovered_{i}}{n_{i}}\;$$

where FI is the individual feed intake (g) in the period, m is the number of days in the period, FeedProvided is the amount of feed provided (g) in the tank, FeedRecovered is the amount of feed recovered (g) in the tank, and n is the number of fish in the tank.

Relative feed intake (RFI)

(4) $$RFI=\;\frac{\frac{FI}{\sqrt{IBW\;\times FBW}}}{d}\times \;100\;$$

where RFI is the relative feed intake (% weight/day) in the period, FI is the individual feed intake (g/fish) in the period, IBW is the initial body weight (g), FBW is the final body weight (g) and d is the number of days in the period.

Feed conversion ratio (FCR)

(5) $$FCR=\frac{FI}{WG}\;$$

where FCR is the feed conversion ratio (g feed/g weight gain), FI is the individual feed intake (g) in the period and WG the weight gain (g) in the period.

Condition factor (K)

(6) $$K=\left(\frac{W}{L^{3}}\right)\times \;100\;$$

where K is the condition factor, W is the fish weight (g) and L is the fork length (cm).

Hepatosomatic Index (HSI)

(7) $$HSI=\left(\frac{LW}{W}\right)\times \;100$$

where HSI is the hepatosomatic index (%), LW is the liver weight (g) and W is the fish weight (g).

Cardiosomatic Index (CSI)

(8) $$CSI=\left(\frac{HW}{W}\right)\times \;100$$

where CSI is the cardiosomatic index (%), HW is the heart weight (g) and W is the fish weight (g).

Gonadosomatic Index (GSI)

(9) $$GSI=\left(\frac{GW}{W}\right)\times \;100\;$$

where GSI is the gonadosomatic index (%), GW is the gonad weight (g) and W is the fish weight (g).

Viscerosomatic Index (VSI)

(10) $$VSI=\left(\frac{VW}{W}\right)\times \;100$$

where VSI is the viscerosomatic index (%), VW is the viscera weight (g) and W is the fish weight (g).

The equations described by Garcia and Gordon (1992) [9] were used to convert from dissolved oxygen saturation (%) to concentration (mg/L).

### 4.5 Tail and opercular beat frequency

The tail and operculum beat frequency are frequently utilized as indicators of oxygen consumption, metabolic rate and overall fish activity level, owing to their direct correlation with energy expenditure adjustments across varying environmental conditions [10,11].

In Trial A exclusively, video footage was captured using GoPro cameras securely mounted atop tank lids to maintain an unobtrusive recording setting. Each tank underwent uninterrupted recording for 15 min while the fish remained undisturbed. The recorded footage underwent thorough examination on a computer screen, leveraging slow-motion playback to enable meticulous analysis. To ensure comprehensive evaluation, a systematic methodology was employed. Ten distinct individuals per tank were intentionally chosen from different spatial locations within the tank. The quantification of tail and opercular beat frequency entailed visually tallying the number of beats within a standardized 20-s interval.

### 4.6 Statistical analysis

Aggregated data by treatment are expressed as mean ± standard deviation. All statistical analysis was performed in the R software. Data was transformed whenever necessary (arcsine and log), checked for normality (the ‘skewKurt’ function from *nipnTK* package was used; z-scores for both skewness and kurtosis inferior to 1.96 indicated normality) and homoscedasticity (based on the Levene's test, for which the ‘leveneTest’ function from the *car* package was used), and further tested through classic One-Way ANOVA (for cases where normality and homoscedasticity were met, for which the ‘oneway.test’ function from the *stats* package with the ‘var.equal’ argument set to TRUE was used) or Welch's ANOVA (for cases where normality and homoscedasticity were not met, for which the ‘oneway.test’ function from the *stats* package with the ‘var.equal’ argument set to FALSE was used), followed by *post-hoc* testing (Tukey's test, in case of One-way ANOVA, for which the ‘TukeyHSD’ function from the *stats* package was used; or Games-Howell test, in case of Welch's ANOVA, for which the ‘games_howell_test’ function from the *rstatix* package was used) to identify which treatments differed from each other, considering α = 0.05. All statistical analysis was performed in the R software.

## 5 Limitations

### 5.1 The main limitations of this dataset are

• Incomplete feed intake records: Time series data for feed intake are missing for some days (was not recorded during most public holidays and weekends).
• Initial weight estimation: The initial average weight of the fish was estimated based on bulk-weighing of the entire population, lacking individual weight variation data for each tank. We suspect significant individual weight variation among the initial fish population, which likely reduced the representativeness of some subsequent samples.
• Short trial period: Although the trials lasted one month, this time period may not be sufficient to robustly assess the impact of different oxygen levels on certain zootechnical indicators.

## Ethics Statement

This dataset includes data collected from *in vivo* animal trials. All experiments complied with the ARRIVE guidelines [12] and were carried out in accordance with the U.K. Animals (Scientific Procedures) Act, 1986 and associated guidelines, and EU Directive 2010/63/EU for animal experiments. The sex of animals was recorded whenever it was considered to have an influence on the measured response variables (e.g., gonad weight).

## CRediT authorship contribution statement

**Nina Liland:** Conceptualization, Methodology, Formal analysis, Investigation, Resources, Writing – review & editing, Supervision, Project administration. **Ivar Rønnestad:** Conceptualization, Methodology, Investigation, Writing – review & editing. **Marina Azevedo:** Formal analysis, Investigation, Data curation, Writing – review & editing. **Floriana Lai:** Investigation, Data curation. **Frida Oulie:** Investigation, Data curation. **Luís Conceição:** Conceptualization, Writing – review & editing, Funding acquisition. **Filipe Soares:** Conceptualization, Methodology, Software, Formal analysis, Data curation, Writing – original draft, Visualization, Project administration.

## Declaration of Competing Interest

The authors declare that they have no known competing financial interests or personal relationships that could have appeared to influence the work reported in this paper.

## Data Availability

Dataset - Atlantic salmon performance at different dissolved oxygen levels (Original data) (Mendeley Data).
